# Targeting Mitochondrial Fission Using Mdivi-1 in A Clinically Relevant Large Animal Model of Acute Myocardial Infarction: A Pilot Study

**DOI:** 10.3390/ijms20163972

**Published:** 2019-08-15

**Authors:** Sang-Bing Ong, Xiu-Yi Kwek, Khairunnisa Katwadi, Sauri Hernandez-Resendiz, Gustavo E. Crespo-Avilan, Nur Izzah Ismail, Ying-Hsi Lin, En Ping Yap, Song-Yi Lim, K P Myu Mai Ja, Chrishan J.A. Ramachandra, Nicole Tee, Jin Jiat Toh, Winston Shim, Philip Wong, Hector A. Cabrera-Fuentes, Derek J Hausenloy

**Affiliations:** 1 Cardiovascular and Metabolic Disorders Program, Duke-National University of Singapore Medical School, Singapore 169857, Singapore; 2 Department of Cardiovascular, Renal and Metabolic Medicine, School of Medicine, Sapporo Medical University, Hokkaido 060-8543, Japan; 3 National Heart Research Institute Singapore, National Heart Centre, Singapore 169609, Singapore; 4 Institute of Biochemistry, Medical School, Justus-Liebig University, 35392 Giessen, Germany; 5 Innoheart Pte Ltd., Singapore 119844, Singapore; 6 Health and Social Sciences Cluster, Singapore Institute of Technology, Singapore 138683, Singapore; 7 Tecnologico de Monterrey, Centro de Biotecnologia-FEMSA, Monterrey, NL 64849, Mexico; 8 Institute of Fundamental Medicine and Biology, Kazan (Volga Region) Federal University, 420008 Kazan, Russian; 9 Yong Loo Lin School of Medicine, National University Singapore, Singapore 119228, Singapore; 10 The Hatter Cardiovascular Institute, Institute of Cardiovascular Science, University College London, London WC1E 6HX, UK; 11 The National Institute of Health Research University College London Hospitals Biomedical Research Centre, London W1T 7DN, UK

**Keywords:** mdivi-1, mitochondrial morphology, cardioprotection, Drp1, pig, ischemia/reperfusion injury

## Abstract

**Background**: New treatments are needed to reduce myocardial infarct size (MI) and prevent heart failure (HF) following acute myocardial infarction (AMI), which are the leading causes of death and disability worldwide. Studies in rodent AMI models showed that genetic and pharmacological inhibition of mitochondrial fission, induced by acute ischemia and reperfusion, reduced MI size. Whether targeting mitochondrial fission at the onset of reperfusion is also cardioprotective in a clinically-relevant large animal AMI model remains to be determined. **Methods**: Adult pigs (30–40 kg) were subjected to closed-chest 90-min left anterior descending artery ischemia followed by 72 h of reperfusion and were randomized to receive an intracoronary bolus of either mdivi-1 (1.2 mg/kg, a small molecule inhibitor of the mitochondrial fission protein, Drp1) or vehicle control, 10-min prior to reperfusion. The left ventricular (LV) size and function were both assessed by transthoracic echocardiography prior to AMI and after 72 h of reperfusion. MI size and the area-at-risk (AAR) were determined using dual staining with Tetrazolium and Evans blue. Heart samples were collected for histological determination of fibrosis and for electron microscopic analysis of mitochondrial morphology. **Results**: A total of 14 pigs underwent the treatment protocols (eight control and six mdivi-1). Administration of mdivi-1 immediately prior to the onset of reperfusion did not reduce MI size (MI size as % of AAR: Control 49.2 ± 8.6 vs. mdivi-1 50.5 ± 11.4; p = 0.815) or preserve LV systolic function (LV ejection fraction %: Control 67.5 ± 0.4 vs. mdivi-1 59.6 ± 0.6; p = 0.420), when compared to vehicle control. Similarly, there were no differences in mitochondrial morphology or myocardial fibrosis between mdivi-1 and vehicle control groups. **Conclusion**: Our pilot study has shown that treatment with mdivi-1 (1.2 mg/kg) at the onset of reperfusion did not reduce MI size or preserve LV function in the clinically-relevant closed-chest pig AMI model. A larger study, testing different doses of mdivi-1 or using a more specific Drp1 inhibitor are required to confirm these findings.

## 1. Introduction

Acute myocardial infarction (AMI) and the heart failure that often follows are among the leading causes of death and disability worldwide [1,2,3,4,5]. As such, novel treatments are needed to reduce myocardial infarct (MI) size and preserve the left ventricular (LV) function, in order to improve health outcomes following AMI [6,7,8,9,10,11,12]. Despite timely reperfusion by primary percutaneous coronary intervention (PPCI), morbidity and mortality remain significant following AMI with 7% death and 22% re-hospitalization for heart failure rates over one year in patients with larger myocardial infarcts [13]. One contributing factor is the existence of “myocardial reperfusion injury”, which refers to the myocardial injury and cardiomyocyte death that occurs on reperfusion of acutely ischemic myocardium and is due to a number of factors, including mitochondrial calcium overload, mitochondrial oxidative stress, ATP depletion, and the opening of the mitochondrial permeability transition pore (MPTP) [14,15,16,17,18,19]. 

Mitochondria are dynamic organelles that can interchange their shape between elongated and fragmented phenotypes through mitochondrial fusion and fission, respectively, which are processes that are governed by mitochondrial shaping proteins [20,21,22,23,24,25,26]. The mitochondrial fission protein, dynamin-related protein 1 (Drp1), mediates the division of mitochondria, which is required to distribute mitochondria during cellular proliferation and is needed for the selective removal of damaged mitochondria by mitophagy [27,28,29,30,31]. A number of experimental studies have demonstrated that mitochondria undergo Drp-1-mediated fission to generate fragmented mitochondria during acute myocardial IR, resulting in mitochondrial dysfunction and cardiomyocyte death, and genetic or pharmacological inhibition of mitochondrial fission in this setting have been reported to reduce MI size in rodent AMI models [32,33,34,35,36,37,38,39,40,41,42,43,44,45,46]. Importantly, studies have shown that targeting mitochondrial fission specifically at reperfusion using either mdivi-1 (a small molecule inhibitor of Drp1) [40,47] or P110 (a peptide inhibitor of the interaction between the mitochondrial fission proteins Drp1 and hFis1) [41] were also able to reduce the MI size in rodent AMI models, demonstrating the clinical applicability of this therapeutic approach. The next step in the clinical translation pathway is to demonstrate the efficacy in a clinically relevant large animal AMI model. Therefore, in the current study, we investigated the cardioprotective effect of mdivi-1 administered as an intracoronary bolus at the onset of reperfusion in a pig model of AMI. 

## 2. Results

A total of 18 pigs (11 vehicle control and seven mdivi-1) underwent the research protocol, with 14 animals (eight vehicle control and six mdivi-1) surviving a three day reperfusion period, and four animals (three vehicle control and one mdivi-1) dying during the ischemic period due to fatal ventricular fibrillation, thus indicating a survival rate of 78%. There were no differences in non-fatal ventricular arrhythmias, heart rate, and oxygen saturations between the vehicle control and mdivi-1 treated animals (Table 1). 

### 2.1. Mdivi-1 Treatment Had No Effect on Post-AMI Mitochondrial Morphology or Myocardial Fibrosis

**Figure 1:**
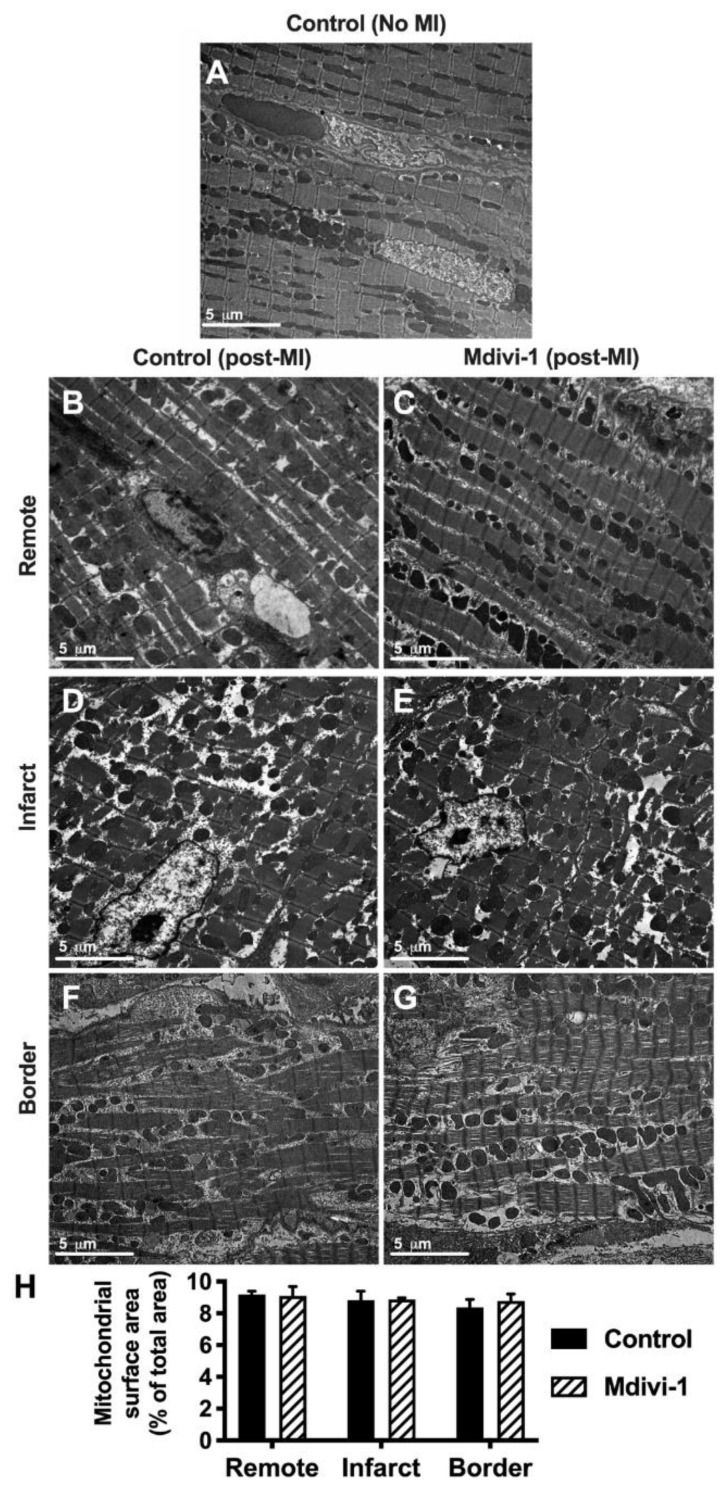
Representative electron microscopy images in: (**A**) Non-infarcted heart; (**B**, **D**, **F**) infarcted hearts administered vehicle control at reperfusion in remote, infarct, and border zones, respectively; and (**C**, **E**, **G**) infarcted hearts administered mdivi-1 at reperfusion in remote, infarct, and border zones, respectively. (**H**) Mitochondrial surface area as a percentage of image area in infarcted hearts. Data are presented as mean ± SD.

We investigated the effect of administering mdivi-1 at the onset of reperfusion on mitochondrial morphology using electron microscopy (Figure 1). With acute myocardial ischemia-reperfusion injury (IRI), there was myofibril disarray and rounding of mitochondria within the infarct and border zones in both vehicle control and mdivi-1 treated animals, when compared to the control heart in the absence of IRI. However, there were no significant differences in the mitochondrial area in remote, border, or infarct zones between the vehicle control and mdivi-1 treatment groups following AMI. 

**Figure 2:**
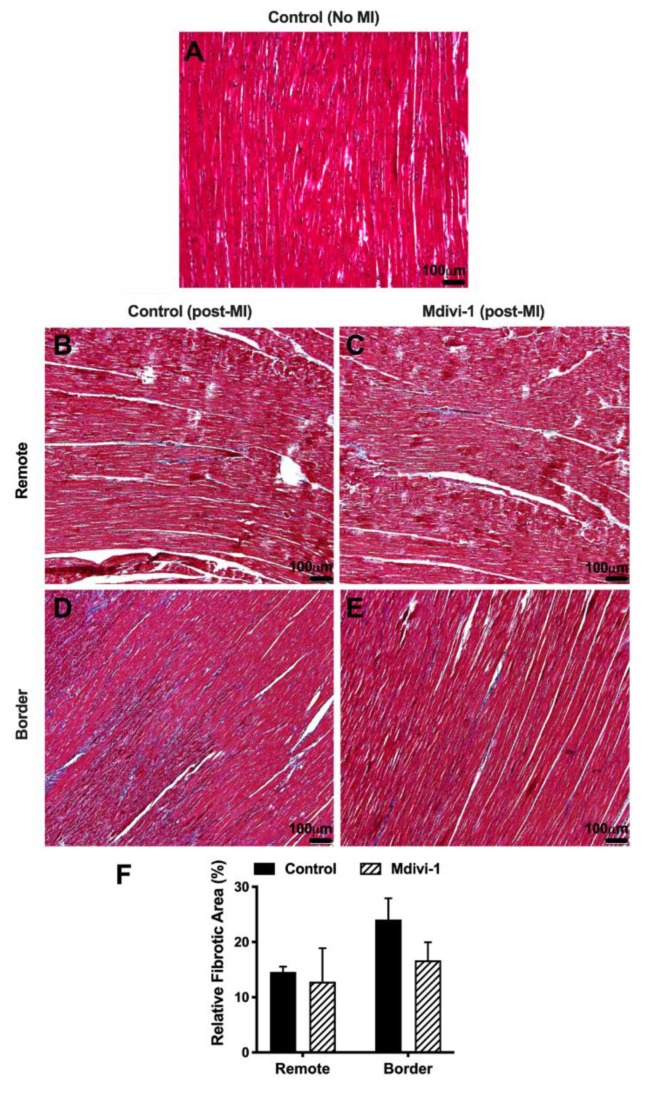
Representative histological images in: (**A**) Non-infarcted heart; (**B**, **D**) infarcted hearts administered vehicle control at reperfusion in remote and border zones, respectively; and (**C**, **E**) infarcted hearts administered mdivi-1 at reperfusion in remote and border zones, respectively. (**F**) Fibrosis quantification in the infarcted heart sections of vehicle control or mdivi-1 treated pigs in the remote and border zones. Data are presented as mean ± SD.

In addition, there was no difference in the extent of myocardial fibrosis in the border zone or remote myocardium when the mdivi-1 and vehicle control treatment groups were compared (Figure 2). 

### 2.2. Mdivi-1 Treatment Had No Effect on Cardiac Size or Function

**Figure 3:**
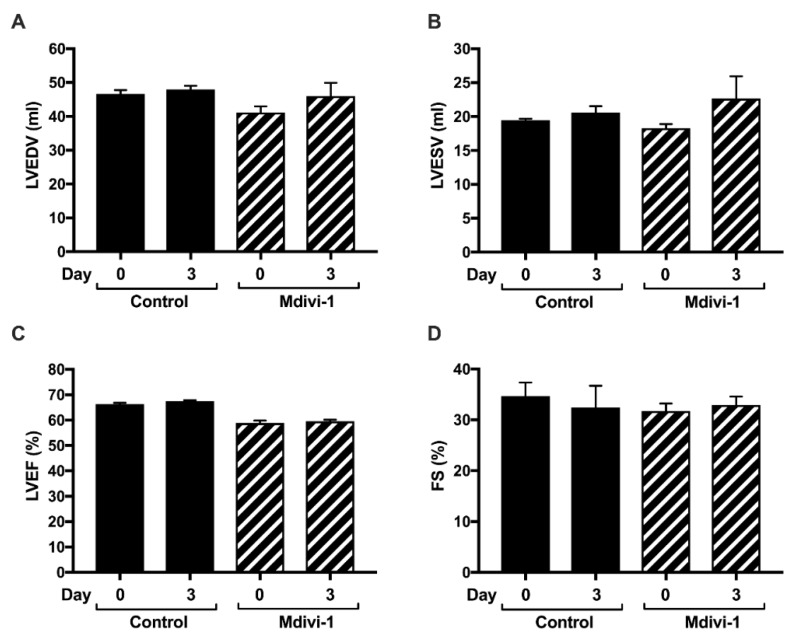
Echocardiographic evaluation of LV size and function prior to AMI and three days post-AMI. (**A**) LVEDV, left ventricular end diastolic volume; (**B**) LVESV, left ventricular end systolic volume; (**C**) LVEF, left ventricular ejection fraction; and (**D**) FS, fractional shortening. There were no significant differences in any of these parameters between vehicle control (*n* = 8) and mdivi-1 (*n* = 6) treated pigs. Data are presented as mean ± SEM.

Next, we investigated whether mdivi-1 treatment at reperfusion had any effect on the LV size and function following AMI, and was assessed by transthoracic echocardiography. There were no significant differences in the LV chamber size (LVEDV and LVESV), LV ejection fraction, or fractional shortening between mdivi-1 and vehicle control treatment groups after 72 h of reperfusion post-AMI (Table 2 and Figure 3).

### 2.3. Mdivi-1 Treatment Did Not Reduce MI Size

**Figure 4:**
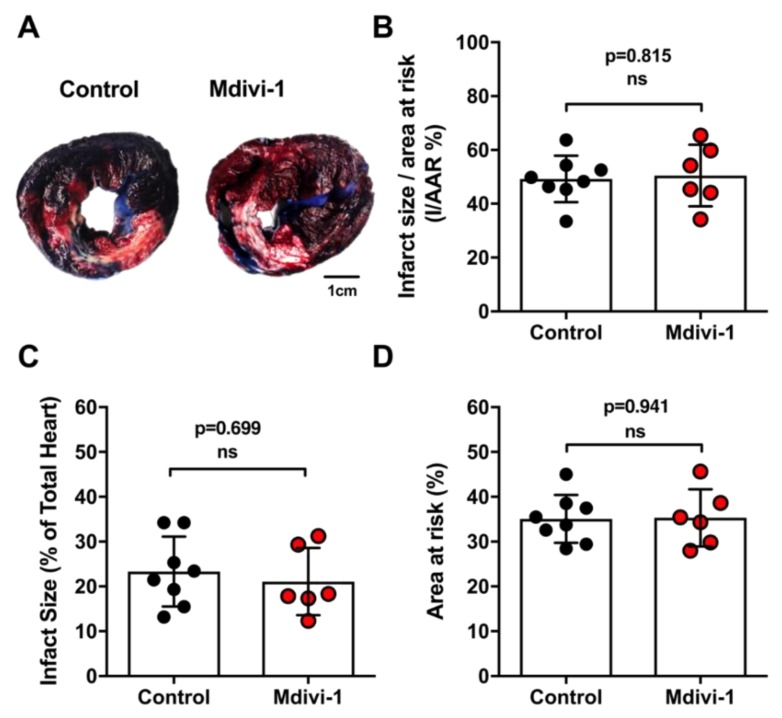
Myocardial infarct in vehicle control and mdivi-1 treatment groups. (**A**) Representative images of TTC/Evans Blue-staining of heart sections from vehicle control or mdivi-1-treated pigs. (**B**) Infarct size over area-at-risk; (**C**) infarct size over total heart volume; and (**D**) area-at-risk. There were no significant differences in any of these parameters between vehicle control (*n* = 8) and mdivi-1 (*n* = 6) treated pigs. Data are presented as mean ± SD.

There were no significant differences in MI size when expressed either as % AAR with 49.2 ± 8.6% in control versus 50.5 ± 11.4% in mdivi-1 (*p* = 0.815) or as % of whole heart with 23.3 ± 7.8% in control versus 21.7 ± 7.4% with mdivi-1 (*p* = 0.699) (Figure 4A–C). There was also no difference in the size of the AAR between the two treatment groups (35.4 ± 5.3% in control versus 35.3 ± 6.3% in mdivi-1; *p* = 0.942) (Figure 4D). 

## 3. Discussion

This study investigated for the first time the effects of targeting mitochondrial fission at the onset of reperfusion in a clinically relevant pig AMI model. This is a critical step in the translation of a novel cardioprotective agent from the laboratory to the clinical setting. We found that a single intracoronary bolus of mdivi-1*,* a small molecule inhibitor of Drp1, did not reduce the MI size or preserve the LV function following AMI, when compared to vehicle control. 

A number of previous experimental studies have demonstrated that mitochondria undergo fission to generate fragmented mitochondria during acute myocardial IRI, and genetic or pharmacological inhibition of mitochondrial fission in this setting were reported to reduce the MI size in rodent AMI models [32,33,36,37,38,39,40,41,42,43,44,45]. Interestingly, targeting mitochondrial fission has been shown to protect other organs such as the kidney [48] and brain [49,50,51,52,53] against detrimental effects of acute IRI. In an earlier study, we demonstrated that mitochondria undergo fission in response to acute myocardial IRI and that pre-treatment with mdivi-1, a small molecule inhibitor of Drp1, inhibited mitochondrial fission and reduced cell death in adult murine cardiomyocytes subjected to simulated IRI, and reduced MI size in an in vivo murine AMI model [32]. In that study, the cardioprotective effect of mdivi-1 was linked to the suppression of MPTP opening, a critical determinant of cardiomyocyte death following acute IRI. However, the mechanisms though which inhibiting mitochondrial fission prevents MPTP opening at reperfusion are not clear, but it may be due to attenuating mitochondrial calcium overload and oxidative stress and preserving mitochondrial respiration. An interesting recent study has shown that inhibiting mitochondrial fission using mdivi-1 was able to reduce mitochondrial fission and cardiac dysfunction induced by acute renal IRI, suggesting the beneficial effects of this therapeutic approach in the cardio-renal syndrome [54]. Importantly, subsequent studies have showed that targeting mitochondrial fission specifically at reperfusion using either mdivi-1 [40,47] or P110 (a peptide inhibitor of interaction between the mitochondrial fission proteins Drp1 and hFis1) [41] was also able to reduce the MI size in rodent AMI models, demonstrating the clinical applicability of this therapeutic approach. The next step in the pathway to translating a cardioprotective agent into the clinical setting is to demonstrate the efficacy in a clinically-relevant large animal AMI model. A pig’s heart, as a clinically-relevant large animal model, is more comparable to a human heart than a rodent’s heart with regards to its large heart/body weight, lack of coronary collaterals, and similarities in the excitation-contraction coupling as well as β-MHC expression levels [55]. Rodent hearts are adapted to function at very high heart rates with different cardiac action potentials [56]. 

Unfortunately, we were unable to demonstrate MI size reduction or the preservation of the LV function with the administration of mdivi-1 immediately prior to reperfusion in the pig AMI model when compared to vehicle control. Furthermore, we showed no effect of mdivi-1 on either mitochondrial morphology (assessed by electron microscopy) or myocardial fibrosis (assessed by histology). However, it must be noted that three days of reperfusion may have been too early to observe any differences in interstitial fibrosis following AMI in the pig heart. Although we did not observe a significant reduction in LV ejection fraction and fractional shortening in our pig AMI model, we observed substantial MI sizes of 50% of the area-at-risk, suggesting that a significant amount of acute myocardial IRI occurred. 

Potential reasons for the lack of cardioprotection with mdivi-1 in our pig AMI model are discussed below. 

(1) Dosing: In our study, we used a single intracoronary bolus of 1.2 mg/kg mdivi-1, which was the dose used in most rodent AMI [32,38,40] and stroke [49] studies. However, an adjustment of the dose for a cardioprotective agent when moving from a small to a large animal AMI model is not clear, and an alternative dose may have been more effective; 

(2) Delivery: Insufficient delivery of mdivi-1 into the ischemic myocardium in the first few minutes of reperfusion may have been an issue. In this regard, it has been shown that the nanoparticle delivery of mdivi-1 to the ischemic heart enhanced its cardioprotective effect in terms of MI size reduction in the murine AMI model [47], and it would be interesting to test whether mdivi-1 delivered by nanoparticles could reduce MI size in our pig AMI model;

(3) Route of administration: In our pig AMI study, we administered mdivi-1 as a single intracoronary bolus, in order to replicate the clinical setting of reperfusion by primary percutaneous coronary intervention (PPCI) in AMI patients, and to provide a localized delivery of the agent to the ischemic area-at-risk. However, in prior rodent AMI studies, pharmacological inhibition of mitochondrial fission was achieved by systemic IV administration of the cardioprotective agent. The administration of mdivi-1 via the intracoronary artery would be expected to achieve higher tissue concentrations of the drug at the ischemic heart, when compared to giving it as an IV bolus;

(4) The agent: Recent studies have shown that mdivi-1 may have off-target effects on mitochondrial function [57,58], which may have attenuated the cardioprotective effects of this agent when administered at reperfusion in our pig AMI model. A more specific pharmacological inhibitor of mitochondrial fission, such as P110, a peptide inhibitor of the Drp1-hFis1 interaction, may have been more effective in our pig AMI model. A potential limitation of this study is that we did not determine the effect of mdivi-1 on levels of the pro-fission protein Drp1 and its enzymatic activity, as well as other mitochondrial-shaping proteins;

(5) Sample size: Our pilot pig AMI study had a relatively small sample size (eight in vehicle control and six in mdivi-1), although we did not see any differences in MI size with mdivi-1 at the dose of 1.2 mg/kg.

## 4. Materials and Methods

### 4.1. Animal Preparation

All animal protocols were reviewed and approved by the Innoheart Pte Ltd. Institutional Animal Care and Use Committee (IACUC) No INH2017/013, Study Plan No. SP17F-NHCMDV, approval 22 August 2017. Female mixed breed swines, *Sus scrofa* (30–40 kg) were purchased from Culindo Livestock Pte Ltd (Singapore). Animals were prepared for the procedures as per Innoheart Pte Ltd (Singapore), following standard operating procedures (SOP) and IACUC protocol. Information relevant to surgical preparation and anesthesia for the study animal was documented in the animal’s record. The study animal was weighed, anesthetized, instrumented, and monitored in accordance with Innoheart SOP and IACUC protocols.

### 4.2. Echocardiography

At day 0 (prior to AMI) and day 3 (post-AMI), the LV function was assessed by two-dimensional transthoracic echocardiography (TTE). Standard M-mode analysis was performed at the appropriate views to determine LV parameters. Measurements of maximal LV long-axis lengths (L) and endocardial area tracings (a), using the leading-edge method, was performed from digital images captured on cine loops. Left ventricular end-diastolic volume (LVEDV) and left ventricular end-systolic volume (LVESV) were calculated using the single plane area–length method: V = 8 × a^2^/(3 × π × L). Left ventricular ejection fraction (LVEF) was then computed from the formula: LVEF = (LVEDV-LVESV)/LVEDV. The LV-end diastolic diameter (LVEDD) and LV-end systolic diameter (LVESD) were also measured to calculate fractional shortening (FS) using the formula [(LVEDD-LVESD)/LVEDD] × 100. All TTE results were recorded in study-specific case report forms [59]. All TTE analyses were performed by study team members blinded to the treatment allocation.

### 4.3. Vascular Catheter Insertion at Day 0

Cut-down was performed in the neck region to isolate the external jugular vein. A vascular catheter was inserted into the vein and tunneled subcutaneously to the dorsum of the neck. The catheter was aspirated to check for positive blood return in order to confirm patency. If blood return was not present or resistance was met, the catheter was flushed with heparinized saline, the catheter was repositioned, and aspiration test repeated. An appropriate dosage of anticoagulant was administered to maintain catheter patency. The muscles and fascia were closed in a continuous/ interrupted manner using absorbable sutures. The skin was closed in an interrupted manner using non-absorbable sutures.

### 4.4. Pig Model of Acute Myocardial Ischemia/Reperfusion Injury 

The animal was placed in a supine position. Surgical approaches were made from the femoral artery and a 6–8Fr sheath was inserted. A single bolus of Heparin (80–120 IU/kg body weight) was administered prior to coronary angiography. A 6–8Fr guiding catheter was introduced through the sheath to the left main coronary artery under fluoroscopic guidance. An angiogram was performed to locate the LAD. A percutaneous transluminal coronary angioplasty (PTCA) balloon was inserted and positioned at the mid-LAD. The distance from the D1 to the proximal edge of the angioplasty balloon was measured. The balloon was inflated for 90 min to occlude all flow beyond the mid-LAD (TIMI 0 flow). At the 80-min mark of the occlusion, the pigs were randomly assigned to receive either mdivi-1 (1.2 mg/kg) (dissolved in DMSO, 0.1 mL of 0.1%, then diluted in 9 mL of normal saline with 1 mL of contrast to make up 10 mL in volume) or vehicle control, by an intracoronary bolus, through the inner lumen of the balloon, distal to the occlusion. Both types of injections were followed by 5 mL of normal saline flush. An angiogram was performed to ensure the proper injection of the treatments. At the end of the 90-min occlusion, the PTCA balloon was deflated and withdrawn. Additional angiographic images of coronary arteries were obtained to ensure patency of the vessels. The angioplasty devices were withdrawn. The fascia and muscle were then closed in a continuous manner using 2/0 absorbable sutures. The skin was closed in an interrupted manner using 2/0 or 3/0 non-absorbable sutures. The animal was recovered from anesthesia. 

### 4.5. Day 3 Follow-Up and Sacrifice

On Day 3, a follow-up TTE was performed. A right carotid cut-down was performed and a sheath inserted. A guiding catheter was inserted into the coronary artery for blood collection. Thoracotomy was performed and LAD ligated (at the same location with reference to Day 0 balloon occlusion). The ascending aorta and inferior vena cava were identified and the aortic clamp was pre-positioned. The pigtail catheter was placed in the LV cavity. A large incision was made in the IVC to allow the blood to flow out and the pre-positioned aortic clamp on the ascending aorta was quickly clamped, avoiding the trachea and other parts of the heart. A total of 50 mL of 1% Evans Blue was injected into the LV cavity via pigtail catheter to allow Evans Blue to perfuse coronary arteries and render the heart blue. The pig was then euthanized with IV Pentobarbitol. Following asystole, the heart was removed and washed immediately with cold normal saline. The heart was sliced from apex to above ligature, each slice at 1 cm in width. Only slice 3 was 0.5 cm in width (for electron microscopy and histology purposes). Slices were photographed and incubated in 1% tetrazolium trichloride (TTC) (Sigma-Aldrich Chemicals, Zwijndrecht, the Netherlands) at 37 °C in 0.9% NaCl for 10 min to discriminate between infarct tissue and viable myocardium. After incubation, slices were again photographed. The remote area, area-at-risk (AAR), and infarct area were quantified using ImageJ software (NIH, Bethesda, MD, USA) [32,59,60,61,62]. 

### 4.6. Histological Analysis

The infarct zone was identified as TTC-unstained area (light pink to white tissue), the AAR was identified as the TTC-stained area (red tissue), and the border zone was identified as the area between the infarcted and viable myocardium in the AAR. Samples obtained from the heart in the Evan’s blue-stained area opposite to the infarcted area were identified as remote zone. For light microscopic evaluation, heart samples reserved for histology were fixed in 10% formalin. The heart samples were embedded in paraffin and cut into 5-mm-thick sections, mounted on slides, and stained with Masson Trichrome’s stain. Sections were examined under a Leica DFC280 light microscope by Leica Q (DM LB 2/11888110) Win and myocardial fibrosis quantified by Image Analysis System (Leica Micros Imaging Solutions Ltd.; Cambridge, UK). 

### 4.7. Electron Microscopy 

The heart slices reserved for electron microscopy were obtained from the remote, border, and infarct zones as described in Section 4.6. The slices were immersed in fixative containing fresh paraformaldyhyde (1%), glutaraldehyde (1%), CaCl_2_ (0.5 mM), glucose 0.031% in phosphate buffer 0.1 M (pH 7.3), and left overnight before sampling. The heart slice was then post-fixed in OsO_4_ (1%) in phosphate buffer (0.1 M) pH 7.3 at 3.0 °C for 1.5 h. The slice was then washed in phosphate buffer (0.1 M) pH 7.4 and Enbloc stained with 0.5% uranyl acetate in distilled water at 3.0 °C for 30 min.

After rinsing with distilled water, the specimens were dehydrated in a graded ethanol water series and infiltrated with Agar-100 resin overnight (Agar Scientific, Stansted Essex UK). Semi-thin sections were cut at 1µm and mounted on glass slides and stained with toluidine blue (1%) in distilled water for light microscopy. Ultra-thin sections were then cut at 70–80 nm using a diamond knife on a Reichert Ultracut E microtome (Reichert Microscope Services, Buffalo, NY, USA). Sections were collected on 200 mesh copper grids, stained with uranyl acetate and lead citrate. The heart specimens were then viewed and recorded with a Jeol 1010 transition electron microscope (Jeol Ltd., Welwyn Garden City, UK). The assessment of mitochondrial morphology was accomplished by randomly selecting 4 random electron micrographs of longitudinally-arranged cardiomyocytes from the remote, border, and infarct zones of each adult heart, and quantifying mitochondrial surface area using Fiji software (Version 2, Dresden, Germany). 

### 4.8. Statistical Analysis

All values are expressed as mean ± SD or SEM. Data were analyzed by 1-way ANOVA followed by a Tukey multiple-comparison post-hoc test. Differences were considered significant at values of *p* < 0.05.

## 5. Conclusions

Although pharmacological inhibition of the mitochondrial fission protein, Drp1, using either mdivi-1 or P110 at the onset of reperfusion has been reported to reduce MI size in rodent AMI models, in our pilot study we did not find any cardioprotective effect with mdivi-1 (at a dose of 1.2 mg/kg) administered as a single intracoronary bolus in a clinically-relevant pig AMI model. These findings need to be confirmed in a larger study using different doses of mdivi-1 and/or more specific Drp1 inhibitors such as P110. 

## Figures and Tables

**Table 1:** Heart rate and oxygen saturations prior to acute myocardial infarction (AMI) and after reperfusion.

Parameter	Before Myocardial Infarct (MI)		Onset of Reperfusion		120 min after Reperfusion	
	Control	Mdivi-1	Control	Mdivi-1	Control	Mdivi-1
Heart rate (beats per min)	83 ± 7	94 ± 12	82 ± 7	81 ± 16	85 ± 7	76 ± 15
Oxygen Saturation (%)	98.6 ± 0.6	98.5 ± 0.6	97.6 ± 1.1	97.5 ± 1.3	98.8 ± 0.50	98.8 ± 0.4

**Table 2:** Echocardiography parameters prior to AMI and three days post-AMI.

Cardiac Parameters	Vehicle Control Pigs		Mdivi-1 Pigs	
	Day 0	Day 3	Day 0	Day 3
LVEDV (mL)	48.6 ± 1.1	48.0 ± 1.2	41.2 ± 2.1	46.0 ± 4.4
LVESV (mL)	19.5 ± 0.3	20.6 ± 1.0	18.3 ± 0.6	22.7 ± 3.6
LVEF (%)	66.3 ± 0.6	67.5 ± 0.4	58.9 ± 0.9	59.6 ± 0.6
FS (%)	34.7 ± 2.7	32.4 ± 4.3	31.7 ± 1.5	32.9 ± 1.7

*LVEDV*, left ventricular end diastolic volume; *LVESV*, left ventricular end systolic volume; *LVEF*, left ventricular ejection fraction; *FS*, fractional shortening.

## Abbreviations

AMI	Acute myocardial infarction
ATP	Adenosine triphosphate
CVD	Cardiovascular disease
Drp1	Dynamin-related protein 1
ED	End diastolic
ES	End systolic
FS	Fractional shortening
IRI	Ischemia-reperfusion injury
IVS	Interventricular septal wall thickness
LAD	Left anterior descending
LV	Left ventricular
LVEAT	Left ventricular endocardial area tracing
LVEDD	Left ventricular end diastolic diameter
LVEDV	Left ventricular end diastolic volume
LVESD	Left ventricular end systolic diameter
LVESV	Left ventricular end systolic volume
LVEF	Left ventricular ejection fraction
LVLAL	Left ventricular long axis length
LVID	Left ventricular internal diameter
LVPW	Left ventricular posterior wall thickness
Mff	Mitochondrial fission factor
MI	Myocardial infarct
MiD49/51	Mitochondrial dynamics proteins of 49 and 51 kDa
mPTP	Mitochondrial permeability transition pore
PTCA	Percutaneous transluminal coronary angioplasty
ROS	Reactive oxygen species
TTC	Tetrazolium trichloride
